# Real-Time Fluorescence Loop Mediated Isothermal Amplification for the Detection of *Acinetobacter baumannii*

**DOI:** 10.1371/journal.pone.0066406

**Published:** 2013-07-02

**Authors:** Qinqin Wang, Yanbin Zhou, Shaoli Li, Chao Zhuo, Siqi Xu, Lixia Huang, Ling Yang, Kang Liao

**Affiliations:** 1 Department of Respiratory Medicine, First Affiliated Hospital of Sun Yat-sen University, Guangzhou, Guangdong, China; 2 Institute of Respiratory Diseases, Sun Yat-sen University, Guangzhou, Guangdong, China; 3 State Key Laboratory of Respiratory Diseases, Guangzhou Medical College, Guangzhou, Guangdong, China; 4 Department of Clinical Laboratory, First Affiliated Hospital of Sun Yat-sen University, Guangzhou, Guangdong, China; New England Biolabs, Inc., United States of America

## Abstract

**Background:**

Detection of *Acinetobacter baumannii* has been relying primarily on bacterial culture that often fails to return useful results in time. Although DNA-based assays are more sensitive than bacterial culture in detecting the pathogen, the molecular results are often inconsistent and challenged by doubts on false positives, such as those due to system- and environment-derived contaminations. In addition, these molecular tools require expensive laboratory instruments. Therefore, establishing molecular tools for field use require simpler molecular platforms. The loop-mediated isothermal amplification method is relatively simple and can be improved for better use in a routine clinical bacteriology laboratory. A simple and portable device capable of performing both the amplification and detection (by fluorescence) of LAMP in the same platform has been developed in recent years. This method is referred to as real-time loop-mediated isothermal amplification. In this study, we attempted to utilize this method for rapid detection of *A. baumannii*.

**Methodology and Significant Findings:**

Species-specific primers were designed to test the utility of this method. Clinical samples of *A. baumannii* were used to determine the sensitivity and specificity of this system compared to bacterial culture and a polymerase chain reaction method. All positive samples isolated from sputum were confirmed to be the species of *Acinetobacter* by 16S rRNA gene sequencing. The RealAmp method was found to be simpler and allowed real-time detection of DNA amplification, and could distinguish *A. baumannii* from *Acinetobacter calcoaceticus* and *Acinetobacter* genomic species 3. DNA was extracted by simple boiling method. Compared to bacterial culture, the sensitivity and specificity of RealAmp in detecting *A. baumannii* was 98.9% and 75.0%, respectively.

**Conclusion:**

The RealAmp assay only requires a single unit, and the assay positivity can be verified by visual inspection. Therefore, this assay has great potential of field use as a molecular tool for detection of *A. baumannii*.

## Introduction

*Acinetobacter baumannii*, one of the important nosocomial pathogens, is often associated with epidemic outbreaks of infection. The organism is frequently pandrug-resistant and capable of causing substantial morbidity and mortality in patients with severe underlying diseases, both in the hospital and in the community [1]. Rapid identification of this pathogen is critical for the appropriate therapy and outbreak control. Conventional methods, such as a phenotypic system and commercial phenotypic method, have successfully identified most *Acinetobacter* species. However, these methods can not distinguish *Acinetobacter calcoaceticus*, *Acinetobacter baumannii*, *Acinetobacter* genomic species 3 and *Acinetobacter* genomic species 13TU, and require specific media and several days of incubation [2]. DNA- based methods have been successfully applied in the detection of *A. baumannii* [3], [4]. However, these methods have been hampered by inconsistent results with culture-based methods. Moreover, most protocols developed for identification of *A. baumannii* by PCR have used primers complementary to a universal region found in several different bacterial genera, so that DNA from bacteria belonging to other genera might also be amplified [2], [5]. However, as progress is made towards better outbreak control, more sensitive tools will be required in order to detect low level of *A. baumannii*. Therefore, further efforts are needed to develop next generation molecular tools for field use with a goal that such tools can complement, or in some situations, replace the existing molecular methods for *A. baumannii* detection.

The recently developed loop-mediated isothermal amplification (LAMP) method is a powerful innovative gene amplification technique [6]. It is based on autocycling strand displacement DNA synthesis in the presence of *Bacillus stearothermophilus* (*Bst*) polymerase under isothermal conditions. Six primers are used to recognize eight distinct regions on the target sequence [7]. Given that this method does not require a thermocycler or sophisticated training, it has the potential as a molecular diagnostic tool for point-of-care (POC) diagnosis. Indeed, LAMP has been used for the detection of several infectious diseases such as *Mycobacterium tuberculosis* [8], *Plesiomonas shigelloides* [9], severe acute respiratory syndrome coronavirus [10], human immunodeficiency virus-1 [11], and *Plasmodium vivax* [12].

Recently, the LAMP method has been used for the detection of *A. baumannii* targeting the 16S-23S rRNA intergenic spacer (ITS) sequence [13]. The results showed that the LAMP method was more suitable than PCR for rapid detection of *A. baumannii* in clinical samples, especially for infection control purposes. However, Soo et al. [13] could not effectively distinguish *A. baumannii* from *Acinetobacter* genomic species 3 and *Acinetobacter* genomic species 13TU.

Therefore, we designed species-specific primers and attempted the utility of a simple portable device (tube scanner), in which both the amplification platform (heating block) and fluorescent detection unit for end point use (with the ability to acquire real time data) are combined into a single unit for LAMP assay, to detect *A. baumannii.* The method has been used to detect different human-infecting plasmodium species [14]. This method is referred to as real-time loop-mediated isothermal amplification (RealAmp). In this study, we demonstrated the utility of this method for the detection of *A. baumannii* and distinguishing *A. baumannii* from *Acinetobacter* genomic species 3, *Acinetobacter calcoaceticus* by using species-specific primers and comparing it to an automated identification system VITEK 2 system and a polymerase chain reaction (PCR) method. The VITEK 2 system uses a fluorescence-based technology for routine identification of gram-negative bacteria [15].

## Methods

### Ethics Statement

Samples used in this study were obtained from First Affiliated Hospital of Sun Yat-sen University and State Key Laboratory of Respiratory Diseases, Guangzhou Medical College. This study was approved by the Institutional Research Ethics Committee of First Affiliated Hospital of Sun Yat-sen University and State Key Laboratory of Respiratory Diseases, Guangzhou Medical College. Informed written consent was obtained from each subject.

### *A. baumannii* ATCC19606, *Acinetobacter calcoaceticus*, *Acinetobacter genomic species* 3, *Acinetobacter* genomic species 13TU and clinical samples

*A. baumannii* ATCC19606 was kindly provided by Wang [16]. *Acinetobacter calcoaceticus, Acinetobacter genomic species* 3 and *Acinetobacter* genomic species 13TU were kindly provided by Zhou et al. [17]. A. *baumannii* ATCC19606, *Acinetobacter calcoaceticus, Acinetobacter genomic species* 3 and *Acinetobacter* genomic species 13TU were grown overnight in Luria–Bertani broth at 37°C. Pure genomic DNA was extracted from A. *baumannii* ATCC19606 using a TaKaRa MiniBEST Bacterial Genomic DNA Extraction Kit Ver.2.0 (TaKaRa Code: DV810A), and the genomic DNA was subjected to serial 10-fold dilutions in sterilized distilled water to produce concentrations ranging from 100 ng/μL to 1 pg/μL and assess the correlation between time to amplification and amount of target DNA. In addition, the number of colony forming units/mL (CFU/mL) was determined by using plate count. To test the limits of detection of RealAmp, overnight cultures were serially diluted (ten-fold) in Luria–Bertani broth from starting inocula of 1×10^7^ CFU/mL to 10^1^ CFU/mL before DNA extraction. In addition, to evaluate the specificity of RealAmp, DNA extracts from the array of bacterial species were tested (Table 1). One hundred and sixty-two spontaneous early-morning sputa from patients evaluated using light microscopy (WBC ≥25 and epithelia cells <10 per 100×field) were qualified. Ninety sputum samples identified as A. baumannii *positive* and 72 sputum samples as *A. baumannii* negative by VITEK 2 system (BioMérieux Inc, France), were used to assess the RealAmp assay. All positive samples isolated from sputum were confirmed to be the species of *Acinetobacter* by 16S rRNA gene sequencing. A. baumannii *ATCC19606* DNA was used as a positive control. Blood sample DNA from healthy individual was used as a negative control. Each of the molecular tests was carried out and interpreted by independent researchers blinded to the origin of the specimens and the laboratory results. Samples with discrepant results in the 2 tests were retested.

**Table 1:** Strains used and the results of RealAmp assays.

Strains	No. of strains	RealAmp
Gram-negative organisms		
*Acinetobacter baumannii* ATCC19606 ^a^	1	+
*Pseudomonas aeruginosa* ATCC27853 ^a^	1	−
*Escherichia coli* ATCC25922 ^a^	1	−
*Escherichia coli* ATCC35218 ^a^	1	−
*Acinetobacter calcoaceticus* ^b^	1	−
*Acinetobacter* genomic species 13TU ^b^	1	+
*Acinetobacter* genomic species 3 ^b^	1	−
*Acinetobacter junii* ^c^	2	−
*Acinetobacter lwoffii* ^c^	2	−
*Klebsiella pneumoniae* ^c^	1	−
*Enterobacter cloacae* ^c^	1	−
*Hemophilus influenzae* ^c^	1	−
*Pseudomonas maltophilia* ^c^	1	−
*Pseudomonas cepacia Burkholder* ^c^	1	−
*Proteus mirabilis* ^c^	1	−
Gram-positive organisms		
*Staphylococcus aureus* ATCC25923 ^a^	1	−
*Staphlococcus epidermidis* ^c^	1	−
*Staphylococcus haemolyticus* ^c^	1	−
*Enterococcus faecium* ^c^	1	−
*Enterococcus faecalis* ^c^	1	−
*Streptococcus pneumoniae* ^c^	1	−
Fungi		
*Candida albicans* ^c^	1	−
*Candida glabrata* ^c^	1	−
*Candida tropicalis* ^c^	1	−
Total	26	

a: American Type Culture Collection, USA.

b: Department of Respiratory Diseases, First Affiliated Hospital, College of Medicine, Zhejiang University, Hangzhou, Zhejiang, China.

c: State Key Laboratory of Respiratory Diseases, Guangzhou Medical College, Guangzhou, Guangdong, China.

RealAmp: real-time loop-mediated isothermal amplification.

−: Result of RealAmp reaction was negative.

+: Result of RealAmp reaction was positive.

### Design of RealAmp primers

*A. baumannii* contains a *pgaABCD* locus that encodes proteins that synthesize cell-associated poly-β-(1-6)-N-acetylglucosamine (PNAG). Choi et al. [18] found that all 30 clinical *A. baumannii* isolates examined had the *pga* genes by PCR analysis. The primers forward outer primer (F3), backward outer primer (B3), forward internal primer (FIP), backward internal primer (BIP) and loop backward primer (LB) listed in Table 2 for the RealAmp test were designed by targeting the conserved regions of the *pgaD* gene of *A. baumannii* (GeneBank accession numbers: FJ866500, CP003856, CP003500, CP001937, CP002522, CP001921, CP000863, CP001172, CP001182, CU459141, CP000521, NZ_GG704572). The target selection for primer design can be accomplished by using the Primer Explorer (http://Primer Explorer.jp/e/v4_manual/In.

**Table 2:** Sequences of primers F3, B3, FIP, BIP and LB used in the RealAmp assay.

Target	Sequences(5′–')	Size(bp)	Position
F3	CTTCTGTTAATAGGTCTAAGCG	22	238–59
B3	TTAAATACCCCTGCTCATCA	20	426–45
FIP	CGGCGATCATCCCCATGAAAGCTCATTTTAATTTTATGGGCAA	43	261–83, 301–20
BIP	GCTCCGAATAGCTCTGTTGAGTGCTGAGACTTTTGTAATTCTGAT	45	328–49, 387–09
LB	CTGGCCTCACAGTTTATGGTCA	22	352–73

F3: forward outer primer; B3: backward outer primer.

FIP: forward internal primer; BIP: backward internal primer.

LB: loop backward primer; bp: base pair.

dex.html). The primer specificity was checked using the basic local alignment search tool (BLAST) against human DNA and other *Acinetobacter* sequences in the nonredundant GenBank database.

### DNA extraction

DNA was isolated from all the samples using a boiling method as described by Kocagöz et al. [19] with some modifications. Briefly, each sputum sample was liquefied in 3 times volume of 4% NaOH, placed 40 minutes at room temperature after strong vibration. One milliliter of the liquefaction of sputum was moved into 1.5 ml Eppendorf tube, and then centrifuged at 12,000 rpm for 10 minutes and the precipitate collected. The precipitate was washed by one milliliter of 1×TE (Tris-EDTA) buffer once, and then centrifuged at 12,000 rpm for 10 minutes and the precipitate collected. Forty microliters of sterilized distilled water was added to the tube containing the precipitate and mixed. The tube was heated on a heat-block at 100°C for 15 minutes, and then placed on ice for 10 minutes. Finally, the tube was then centrifuged at 12,000 rpm for 10 minutes and the supernatants were collected and used in the RealAmp and PCR assays.

### Conventional PCR

Conventional PCR was performed as described by Choi et al. [18] with some modifications. Reactions were performed according to TaKaRa Taq^TM^ (Code: DR001A ) in 25 μL total volume containing 1×PCR buffer (10 mM Tris-HCl pH 8.3, 50 mM KCl), 0.2 mM of dNTPs each, 1.5 mM MgCl_2_, 400 nM of F3 and B3 primers each, 0.5 units of Taq Polymerase (Takara Co. Ltd., Japan), and 2 μL of DNA as the template. Reaction conditions were set at 94°C for 5 min, followed by 30 cycles of 94°C for 30 s, 52°C for 30 s, 72°C for 45 s, with a final extension at 72°C for 10 min. Five microliters of the PCR product was then analyzed by 2% agarose gel electrophoresis.

### RealAmp Method

The RealAmp method was performed using the commercially available DNA thermostatic amplification kit (Guangzhou Diao Bio-technology Co., Ltd., Guangdong, China) following the manufacturer's instructions. Reactions were performed in 25 μL total volume containing 2×reaction buffer (40 mM Tris-HCl pH 8.8, 20 mM KCl, 16 mM MgSO4, 20 mM (NH4)_2_SO4, 0.2% Tween-20, 0.8M Betaine, 2.8 mM of dNTPs each), 0.5 μL of a 1∶100 dilution SYBR green I (Invitrogen), 0.2 μM of each outer primers of F3 and B3, 1.6 μM of each inner primers of FIP and BIP, 0.8 μM of loop primer of LB, and 8 units of *Bst* polymerase (New England Biolabs, Ipswich, MA). DNA amplification was carried out at 63°C for 60 minutes using the ESE-Quant Tube Scanner which was set to collect fluorescence signals at 30 seconds intervals. The ESE-Quant Tube Scanner used in this study was developed by a company (QIAGEN Lake Constance GmbH, Stockach, Germany). This device has an eight tube holder heating block with adjustable temperature settings and spectral devices to detect amplified product using fluorescence spectra. This equipment weighs about 1 kg with the dimensions 74 mm×178 mm×188 mm. The unit is completely portable and can be operated with a Li-Ion rechargeable power pack without external power supply. A small liquid crystal display (monitor) is available to display the results (as positive or negative) without the need of a computer. However, the device can also be used together with a computer to generate real time amplification plots as the reaction progresses [14].

### Statistics

The sensitivity and specificity of RealAmp method was calculated using both VITEK 2 system and a conventional PCR assay [14]. The percentage specificity and sensitivity were calculated using the formulae shown below:
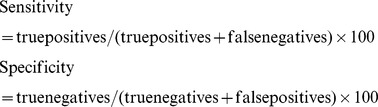



In addition, 95% Confidence Intervals (95%CI) for both sensitivity and specificity were calculated.

## Results

### The correlation between time to amplification and amount of target DNA

**Figure 1:**
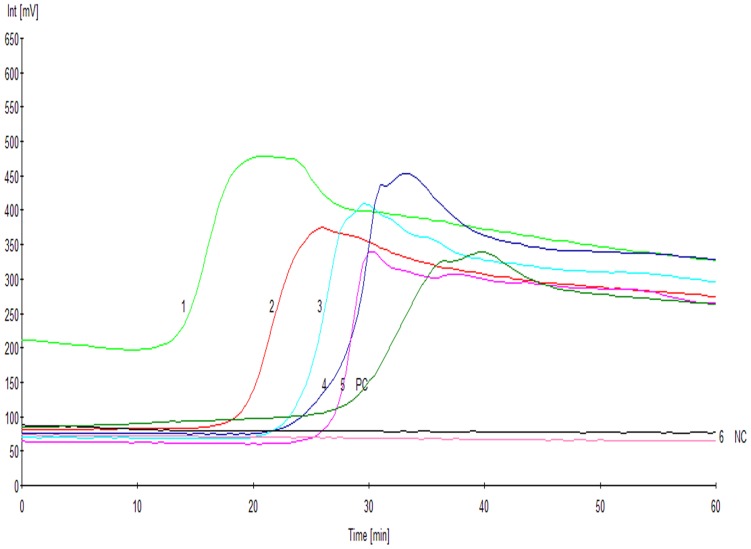
The correlation between time to amplification and amount of target DNA. The plot reported the fluorescence in millivolts (mV) on the Y-axis and time in minutes on the X-axis. 1, 100 ng/μL; 2, 10 ng/μL; 3, 1 ng/μL; 4, 100 pg/μL; 5, 10 pg/μL; 6, 1 pg/μL; PC, positive control; NC, negative control.

The results showed that time to amplification prolonged in line with the decrease of the amount of target DNA (Figure 1).

### Sensitivity of the RealAmp method

**Figure 2.Comparative:**
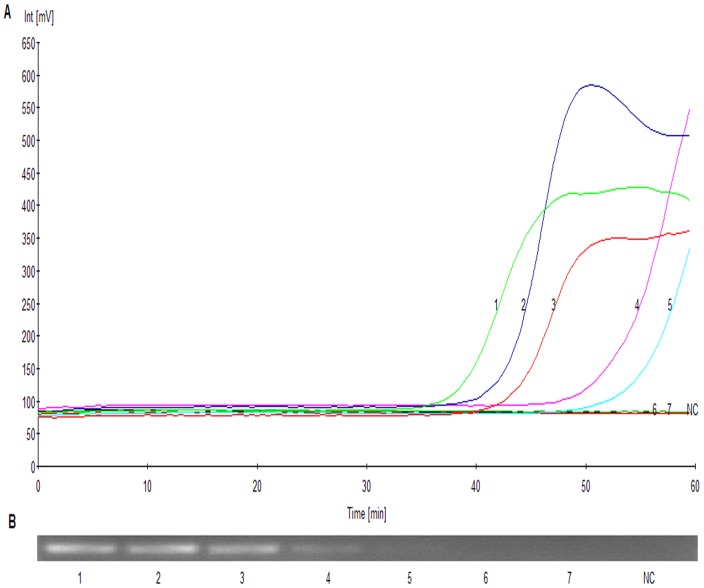
sensitivity of the RealAmp and PCR assays for the detection of *A. baumannii* ATCC19606. (A) The sensitivity of the RealAmp assay for the detection of *A. baumannii* ATCC19606. The plot reported the fluorescence in millivolts (mV) on the Y-axis and time in minutes on the X-axis. 1, 1×10^7^ CFU/mL; 2, 1×10^6^ CFU/mL; 3, 1×10^5^ CFU/mL; 4, 1×10^4^ CFU/mL; 5, 1×10^3^ CFU/mL; 6, 1×10^2^ CFU/mL; 7, 1×10^1^ CFU/mL; NC, negative control. (B) The sensitivity of the PCR assay for the detection of *A. baumannii* ATCC19606. 1, 1×10^7^ CFU/mL; 2, 1×10^6^ CFU/mL; 3, 1×10^5^ CFU/mL; 4, 1×10^4^ CFU/mL; 5, 1×10^3^ CFU/mL; 6, 1×10^2^ CFU/mL; 7, 1×10^1^ CFU/mL; NC, negative control. The number of colony forming units/mL (CFU/mL) of *A. baumannii* ATCC19606 are indicated.

The lower limit of detection of RealAmp determined using DNA obtained from *A. baumannii* ATCC19606 was 1×10^3^ CFU/mL, but the conventional PCR only 1×10^4^ CFU/mL (Figure 2). The time to amplification of RealAmp varied between 15–60 minutes. More time to amplification was required for clinical sputum samples with lower bacterial content.

### Specificity of the RealAmp method

**Figure 3:**
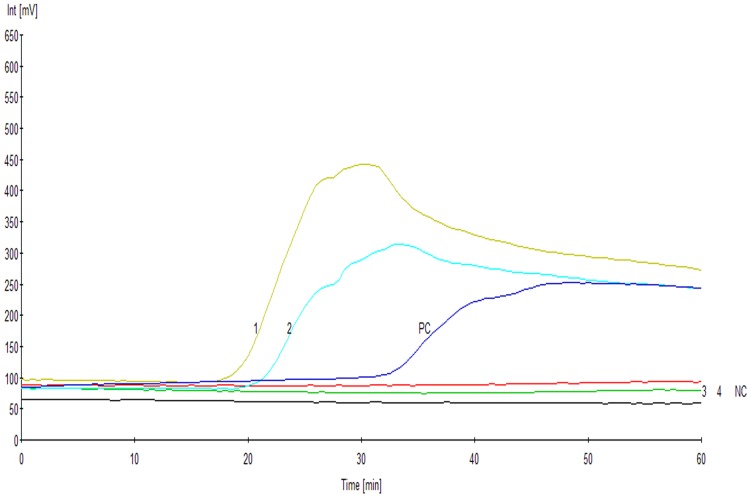
The RealAmp assay for the detection of *A. calcoaceticus–A. baumannii (Ac–Ab)* complex. The plot reported the fluorescence in millivolts (mV) on the Y-axis and time in minutes on the X-axis. 1, *Acinetobacter baumannii* ATCC19606; 2, *Acinetobacter* genomic species 13TU; 3, *Acinetobacter* genomic species 3; 4, *Acinetobacter calcoaceticus*; PC, positive control; NC, negative control.

We selected 6 *Acinetobacter* species (8 strains) and 18 strains belonging to other genera (Table 1). The results showed that the RealAmp assay could effectively differentiate *A. baumannii* from 18 strains of other non-*Acinetobacter* genera (Table 1). In addition, in the detection of the *A. calcoaceticus–A. baumannii (Ac–Ab)* complex, the results showed that the RealAmp assay could differentiate *A. baumannii* from *Acinetobacter calcoaceticus* and *Acinetobacter* genomic species 3 (Figure 3).

### Identification of bacterial strains isolated from clinical sputum samples

Clinical sputum samples were first discriminated by VITEK 2 system and further identified by 16S rRNA gene sequencing, and then assessed by conventional PCR and RealAmp established in this study, with median bacterial content of 1×10^5^ CFU/mL (range 1×10^3^–1×10^8^ CFU/mL) (Table S1). The sensitivity and specificity of the RealAmp method compared to VITEK 2 system and conventional PCR were listed in Table 3. Of the 90 positive samples tested in VITEK 2 system, 89 samples were confirmed to be positive in the RealAmp assay and 84 positive in the PCR assay. Fifty-four out of the 72 negative samples by VITEK 2 system were shown to be negative by RealAmp and 60 negative by the PCR assay. The RealAmp assay showed 98.9% (95% CI: 94.0–99.8%) sensitivity and 75.0% (95% CI: 63.9–83.6%) specificity when compared to VITEK 2 system. The RealAmp method showed 100% (95% CI: 95.6–100%) sensitivity and 90.0% (95% CI: 79.9–5.3%) specificity when compared to the PCR assay. Overall, the sensitivity of conventional PCR and RealAmp was similar, when compared to VITEK 2 system data.

**Table 3:** Sensitivity and Specificity of the RealAmp assay compared to VITEK 2 system and PCR assay.

VITEK 2 system(n)	RealAmp	PCR			
	Positive	Negative	Positive		Negative
Positive (90)*	89	1	84		6
Negative (72)	18*	54	12*		60
Sensitivity	98.9% (95% CI: 94.0–99.8%)		93.3% (95% CI: 86.2–96.9%)		
Specificity	75.0% (95% CI: 63.9–83.6%)		83.3% (95% CI: 73.1–90.2%)		
Positive likelihood ratio	3.956 (95%CI: 2.65–.905)		5.600(95%CI: 3.331–.414)		
Negative likelihood ratio	0.015(95%CI:0.002–.105)		0.080(95%CI:0.037–.174)		
Diagnostic odds ratio	267.000(95%CI:34.653–057.206)		70.000(95%CI:24.879–96.952)		

* : The species of *Acinetobacter* determined by 16S rRNA gene sequencing.

VITEK 2 system: a fluorescence-based automated identification system.

RealAmp: real-time loop-mediated isothermal amplification.

PCR: polymerase chain reaction.

## Discussion

Recently, multiplex PCR and real-time PCR approaches have been found to be useful for detection of *A. baumannii* strains [20], [21]. Multiplex PCR can achieve simultaneous detection of multiple agents that cause similar or identical clinical syndromes and/or share similar epidemiological features in a single test. However, multiplex PCR can pose several difficulties, including poor sensitivity or specificity and/or preferential amplification of certain specific targets. The presence of more than one primer pair in the multiplex PCR increases the chance of obtaining spurious amplification products, primarily because of the formation of primer dimers [20], [22]. Real-time PCR assay have many advantages over conventional PCR, including rapidity, lower contamination rate, higher sensitivity and easy standardization. However, demanding operation and expensive kits and equipment restrict its clinical application [23].

In this study, a rapid RealAmp assay for the detection of *A. baumannii* was developed by using ESE-Quant tube scanner, which is portable, small, lightweight, and can be operated by an optional Li-Ion rechargeable power pack to achieve amplification step and product detection step simultaneously [14]. The RealAmp test does not require time-consuming steps for DNA purification, or downstream processing for amplicon detection. More importantly, the interpretation of results from this closed-tube test does not require highly experienced staff. A small LCD panel is available to display the results (as positive or negative). These features make the RealAmp assay an excellent option for the molecular test of *A. baumannii* even in basic healthcare settings. However, the disadvantage of the device is that it can accommodate only eight samples once and uses nonspecific dye [24]. Therefore, modification is required to expand the device to accommodate more samples.

Due to the high level of genotypic and phenotypic similarity among *Acinetobacter calcoaceticus*, *A. baumannii*, *Acinetobacter* genomic species 13TU and *Acinetobacter* genomic species 3, they are grouped together as the *A. calcoaceticus–A. baumannii (Ac–Ab)* complex [25]. However, there are considerable epidemiological and clinically relevant differences among these species. To our knowledge, *A. calcoaceticus* is an environmental organism, which has never been involved in serious human disease, and therefore it should not be misidentified as *A. baumannii* [26]. The natural habitats of *A. baumannii* and *Acinetobacter* genomic species 13TU are unknown, as are the differences in their epidemic behaviors, resistance mechanisms, and pathogenicities. *Acinetobacter* genomic species 3 can be found regularly on human skin, as well as in aquatic environments [27]. *Acinetobacter* genomic species 3 has also been implicated in nosocomial infections, but its tendency for epidemic spread and resistance development is far less obvious than that of *A. baumannii* [27]. For epidemiological and clinical purposes, it is very critical to differentiate among these species correctly. In our study, the species-specific primers were designed to distinguish *A. baumannii* from *Acinetobacter calcoaceticus* and *Acinetobacter* genomic species 3. However, the previously reported LAMP test based on *A. baumannii* targeting the 16S–23S rRNA intergenic spacer sequence could not effectively distinguish *A. baumannii* from *Acinetobacter* genomic species 3 and *Acinetobacter* genomic species 13TU [13].

The RealAmp assay was at least 10-fold more sensitive than the PCR assay. Therefore, the LAMP assay is more suitable than PCR for rapid detection of *A. baumannii*.

Out of the 90 positive samples detected by VITEK 2 system used in this study, 89 were *A. baumannii* and 1 was *Acinetobacter calcoaceticus* ADP1 by 16S rRNA gene sequencing. Six were negative by conventional PCR and 1 by RealAmp assay. Failure to amplify one sample by RealAmp was *Acinetobacter calcoaceticus* ADP1. Therefore, the results showed that the RealAmp assay could differentiate between *A. baumannii* and *Acinetobacter calcoaceticus*. Failure to amplify these samples by conventional PCR was likely due to low bacterial content. But further studies in different transmission settings are required to evaluate the performance of the RealAmp method in comparison with other routine diagnostic tests.

Of the 72 VITEK 2 system negative samples, 60 were negative by conventional PCR and 54 by RealAmp assay. Eighteen were positive by RealAmp assay, which were further confirmed to be *A. baumannii* by 16S rRNA gene sequencing. Moreover, the slower rate of metabolism of *A. baumannii* could cause weaker fluorescent biochemical reactions in the reaction wells of VITEK 2 ID-GNB cards, and this may cause more discrepant identifications with the VITEK 2 system [28]. Joyanes et al. [15] found that the VITEK 2 system identified 91.6%, 100%, and 76% of *P. aeruginosa*, *S. maltophilia*, and *A. baumannii* isolates, within 3 h. However, the RealAmp assay could detect *A. baumannii* isolates which would not be affected by biochemical reactions. In addition, for the RealAmp assay, the targets for detection are representative sequences from the pathogens, alive or dead. Culture-based assays, on the other hand, detect all living bacterial cells grown in the culture media. To be objective, there are more elements and factors to be considered when culture-based assays are performed, such as how to keep the cells alive and what selective media to use for visualizing the colonies.

The RealAmp method will be more attractive for field use if the LAMP reagents can be made into dry powder and stored at room temperature without requiring a cold chain. In summary, this study has shown that the RealAmp method is a potential of field use as a tool for detection of *A. baumannii*.

## Supporting Information

Table S1 **The information of the clinical samples in this study.**
(DOC)
