# Supplementary material for: Real-Time Fluorescence Loop Mediated Isothermal Amplification for the Detection of Acinetobacter baumannii
Source: PLoS One. 2013 Jul 2;8(7):e66406. doi: 10.1371/journal.pone.0066406 (PMC3699609; doi:10.1371/journal.pone.0066406)
Supplement: Table S1 — The information of the clinical samples in this study. (DOC) [file pone.0066406.s001.doc]

**Table S1.** The information of the clinical samples in this study.

| NO. | Strains* | Results of VITEK 2 system | Results of PCR | Results of RealAmp | Bacterial load of sputum samples (CFU/mL ) |
| --- | --- | --- | --- | --- | --- |
| 1 | *Acinetobacter baumannii* | *Acinetobacter baumannii* | - | + | 10 ^5^ |
| 2 | *Acinetobacter baumannii* | *Acinetobacter baumannii* | - | + | 10 ^6^ |
| 3 | *Acinetobacter baumannii* | *Acinetobacter baumannii* | - | + | 10 ^4^ |
| 4 | *Acinetobacter baumannii* | *Acinetobacter baumannii* | - | + | 10 ^7^ |
| 5 | *Acinetobacter baumannii* | *Acinetobacter baumannii* | - | + | 10 ^6^ |
| 6 | *Acinetobacter baumannii* | *Acinetobacter baumannii* | - | + | 10 ^7^ |
| 7 | *Acinetobacter baumannii* | *Acinetobacter baumannii* | + | + | 10 ^7^ |
| 8 | *Acinetobacter baumannii* | *Acinetobacter baumannii* | + | + | 10 ^7^ |
| 9 | *Acinetobacter baumannii* | *Acinetobacter baumannii* | + | + | 10 ^6^ |
| 10 | *Acinetobacter baumannii* | *Acinetobacter baumannii* | + | + | 10 ^7^ |
| 11 | *Acinetobacter baumannii* | *Acinetobacter baumannii* | + | + | 10 ^7^ |
| 12 | *Acinetobacter baumannii* | *Acinetobacter baumannii* | + | + | 10 ^8^ |
| 13 | *Acinetobacter baumannii* | *Acinetobacter baumannii* | + | + | 10 ^6^ |
| 14 | *Acinetobacter baumannii* | *Acinetobacter baumannii* | + | + | 10 ^7^ |
| 15 | *Acinetobacter baumannii* | *Acinetobacter baumannii* | + | + | 10 ^7^ |
| 16 | *Acinetobacter baumannii* | *Acinetobacter baumannii* | + | + | 10 ^7^ |
| 17 | *Acinetobacter baumannii* | *Acinetobacter baumannii* | + | + | 10 ^7^ |
| 18 | *Acinetobacter baumannii* | *Acinetobacter baumannii* | + | + | 10 ^6^ |
| 19 | *Acinetobacter baumannii* | *Acinetobacter baumannii* | + | + | 10 ^4^ |
| 20 | *Acinetobacter baumannii* | *Acinetobacter baumannii* | + | + | 10 ^5^ |
| NO. | Strains* | Results of VITEK 2 system | Results of PCR | Results of RealAmp | Bacterial load of sputum samples (CFU/mL ) |
| 21 | *Acinetobacter baumannii* | *Acinetobacter baumannii* | + | + | 10 ^8^ |
| 22 | *Acinetobacter baumannii* | *Acinetobacter baumannii* | + | + | 10 ^4^ |
| 23 | *Acinetobacter baumannii* | *Acinetobacter baumannii* | + | + | 10 ^3^ |
| 24 | *Acinetobacter baumannii* | *Acinetobacter baumannii* | + | + | 10 ^6^ |
| 25 | *Acinetobacter baumannii* | *Acinetobacter baumannii* | + | + | 10 ^8^ |
| 26 | *Acinetobacter baumannii* | *Acinetobacter baumannii* | + | + | 10 ^8^ |
| 27 | *Acinetobacter baumannii* | *Acinetobacter baumannii* | + | + | 10 ^7^ |
| 28 | *Acinetobacter baumannii* | *Acinetobacter baumannii* | + | + | 10 ^5^ |
| 29 | *Acinetobacter baumannii* | *Acinetobacter baumannii* | + | + | 10 ^8^ |
| 30 | *Acinetobacter baumannii* | *Acinetobacter baumannii* | + | + | 10 ^8^ |
| 31 | *Acinetobacter baumannii* | *Acinetobacter baumannii* | + | + | 10 ^7^ |
| 32 | *Acinetobacter baumannii* | *Acinetobacter baumannii* | + | + | 10 ^7^ |
| 33 | *Acinetobacter baumannii* | *Acinetobacter baumannii* | + | + | 10 ^8^ |
| 34 | *Acinetobacter baumannii* | *Acinetobacter baumannii* | + | + | 10 ^4^ |
| 35 | *Acinetobacter baumannii* | *Acinetobacter baumannii* | + | + | 10 ^3^ |
| 36 | *Acinetobacter baumannii* | *Acinetobacter baumannii* | + | + | 10 ^7^ |
| 37 | *Acinetobacter baumannii* | *Acinetobacter baumannii* | + | + | 10 ^8^ |
| 38 | *Acinetobacter baumannii* | *Acinetobacter baumannii* | + | + | 10 ^3^ |
| 39 | *Acinetobacter baumannii* | *Acinetobacter baumannii* | + | + | 10 ^6^ |
| 40 | *Acinetobacter baumannii* | *Acinetobacter baumannii* | + | + | 10 ^8^ |
| NO. | Strains* | Results of VITEK 2 system | Results of PCR | Results of RealAmp | Bacterial load of sputum samples (CFU/mL ) |
| 41 | *Acinetobacter baumannii* | *Acinetobacter baumannii* | + | + | 10 ^7^ |
| 42 | *Acinetobacter baumannii* | *Acinetobacter baumannii* | + | + | 10 ^7^ |
| 43 | *Acinetobacter baumannii* | *Acinetobacter baumannii* | + | + | 10 ^3^ |
| 44 | *Acinetobacter baumannii* | *Acinetobacter baumannii* | + | + | 10 ^8^ |
| 45 | *Acinetobacter baumannii* | *Acinetobacter baumannii* | + | + | 10 ^7^ |
| 46 | *Acinetobacter baumannii* | *Acinetobacter baumannii* | + | + | 10 ^4^ |
| 47 | *Acinetobacter baumannii* | *Acinetobacter baumannii* | + | + | 10 ^5^ |
| 48 | *Acinetobacter baumannii* | *Acinetobacter baumannii* | + | + | 10 ^7^ |
| 49 | *Acinetobacter baumannii* | *Acinetobacter baumannii* | + | + | 10 ^8^ |
| 50 | *Acinetobacter baumannii* | *Acinetobacter baumannii* | + | + | 10 ^5^ |
| 51 | *Acinetobacter baumannii* | *Acinetobacter baumannii* | + | + | 10 ^8^ |
| 52 | *Acinetobacter baumannii* | *Acinetobacter baumannii* | + | + | 10 ^7^ |
| 53 | *Acinetobacter baumannii* | *Acinetobacter baumannii* | + | + | 10 ^5^ |
| 54 | *Acinetobacter baumannii* | *Acinetobacter baumannii* | + | + | 10 ^8^ |
| 55 | *Acinetobacter baumannii* | *Acinetobacter baumannii* | + | + | 10 ^6^ |
| 56 | *Acinetobacter baumannii* | *Acinetobacter baumannii* | + | + | 10 ^7^ |
| 57 | *Acinetobacter baumannii* | *Acinetobacter baumannii* | + | + | 10 ^8^ |
| 58 | *Acinetobacter baumannii* | *Acinetobacter baumannii* | + | + | 10 ^7^ |
| 59 | *Acinetobacter baumannii* | *Acinetobacter baumannii* | + | + | 10 ^8^ |
| 60 | *Acinetobacter baumannii* | *Acinetobacter baumannii* | + | + | 10 ^6^ |
| NO. | Strains* | Results of VITEK 2 system | Results of PCR | Results of RealAmp | Bacterial load of sputum samples (CFU/mL ) |
| 61 | *Acinetobacter baumannii* | *Acinetobacter baumannii* | + | + | 10 ^7^ |
| 62 | *Acinetobacter baumannii* | *Acinetobacter baumannii* | + | + | 10 ^6^ |
| 63 | *Acinetobacter baumannii* | *Acinetobacter baumannii* | + | + | 10 ^4^ |
| 64 | *Acinetobacter baumannii* | *Acinetobacter baumannii* | + | + | 10 ^8^ |
| 65 | *Acinetobacter baumannii* | *Acinetobacter baumannii* | + | + | 10 ^8^ |
| 66 | *Acinetobacter baumannii* | *Acinetobacter baumannii* | + | + | 10 ^6^ |
| 67 | *Acinetobacter baumannii* | *Acinetobacter baumannii* | + | + | 10 ^8^ |
| 68 | *Acinetobacter baumannii* | *Acinetobacter baumannii* | + | + | 10 ^8^ |
| 69 | *Acinetobacter baumannii* | *Acinetobacter baumannii* | + | + | 10 ^5^ |
| 70 | *Acinetobacter baumannii* | *Acinetobacter baumannii* | + | + | 10 ^5^ |
| 71 | *Acinetobacter baumannii* | *Acinetobacter baumannii* | + | + | 10 ^8^ |
| 72 | *Acinetobacter baumannii* | *Acinetobacter baumannii* | + | + | 10 ^6^ |
| 73 | *Acinetobacter baumannii* | *Acinetobacter baumannii* | + | + | 10 ^8^ |
| 74 | *Acinetobacter baumannii* | *Acinetobacter baumannii* | + | + | 10 ^8^ |
| 75 | *Acinetobacter baumannii* | *Acinetobacter baumannii* | + | + | 10 ^5^ |
| 76 | *Acinetobacter baumannii* | *Acinetobacter baumannii* | + | + | 10 ^6^ |
| 77 | *Acinetobacter baumannii* | *Acinetobacter baumannii* | + | + | 10 ^5^ |
| 78 | *Acinetobacter baumannii* | *Acinetobacter baumannii* | + | + | 10 ^7^ |
| 79 | *Acinetobacter baumannii* | *Acinetobacter baumannii* | + | + | 10 ^8^ |
| 80 | *Acinetobacter baumannii* | *Acinetobacter baumannii* | + | + | 10 ^5^ |
| NO. | Strains* | Results of VITEK 2 system | Results of PCR | Results of RealAmp | Bacterial load of sputum samples (CFU/mL ) |
| 81 | *Acinetobacter baumannii* | *Acinetobacter baumannii* | + | + | 10 ^6^ |
| 82 | *Acinetobacter baumannii* | *Acinetobacter baumannii* | + | + | 10 ^5^ |
| 83 | *Acinetobacter baumannii* | *Acinetobacter baumannii* | + | + | 10 ^8^ |
| 84 | *Acinetobacter baumannii* | *Acinetobacter baumannii* | + | + | 10 ^7^ |
| 85 | *Acinetobacter baumannii* | *Acinetobacter baumannii* | + | + | 10 ^8^ |
| 86 | *Acinetobacter baumannii* | *Acinetobacter baumannii* | + | + | 10 ^6^ |
| 87 | *Acinetobacter baumannii* | *Acinetobacter baumannii* | + | + | 10 ^7^ |
| 88 | *Acinetobacter baumannii* | *Acinetobacter baumannii* | + | + | 10 ^8^ |
| 89 | *Acinetobacter baumannii* | *Acinetobacter baumannii* | + | + | 10 ^5^ |
| 90 | *Acinetobacter calcoaceticus* ADP1 | *Acinetobacter baumannii* | + | - | 10 ^6^ |
| 91 | ND | *Pseudomonas aeruginosa* | - | - | 10 ^7^ |
| 92 | ND | *Pseudomonas aeruginosa* | - | - | 10 ^6^ |
| 93 | ND | *Pseudomonas aeruginosa* | - | - | 10 ^5^ |
| 94 | ND | *Pseudomonas aeruginosa* | - | - | 10 ^5^ |
| 95 | ND | *Pseudomonas aeruginosa* | - | - | 10 ^7^ |
| 96 | ND | *Pseudomonas aeruginosa* | - | - | 10 ^8^ |
| 97 | ND | *Pseudomonas aeruginosa* | - | - | 10 ^4^ |
| 98 | ND | *Pseudomonas aeruginosa* | - | - | 10 ^5^ |
| 99 | ND | *Pseudomonas aeruginosa* | - | - | 10 ^7^ |
| 100 | ND | *Pseudomonas aeruginosa* | - | - | 10 ^6^ |
| NO. | Strains* | Results of VITEK 2 system | Results of PCR | Results of RealAmp | Bacterial load of sputum samples (CFU/mL ) |
| 101 | ND | *Pseudomonas aeruginosa* | - | - | 10 ^5^ |
| 102 | ND | *Pseudomonas aeruginosa* | - | - | 10 ^8^ |
| 103 | ND | *Pseudomonas aeruginosa* | - | - | 10 ^7^ |
| 104 | ND | *Pseudomonas aeruginosa* | - | - | 10 ^7^ |
| 105 | ND | *Pseudomonas aeruginosa* | - | - | 10 ^6^ |
| 106 | ND | *Escherichia*  *coli* | - | - | 10 ^8^ |
| 107 | ND | *Escherichia*  *coli* | - | - | 10 ^5^ |
| 108 | ND | *Escherichia*  *coli* | - | - | 10 ^4^ |
| 109 | ND | *Escherichia*  *coli* | - | - | 10 ^6^ |
| 110 | ND | *Escherichia*  *coli* | - | - | 10 ^6^ |
| 111 | ND | *Escherichia*  *coli* | - | - | 10 ^5^ |
| 112 | ND | *Escherichia*  *coli* | - | - | 10 ^8^ |
| 113 | ND | *Escherichia*  *coli* | - | - | 10 ^7^ |
| 114 | ND | *Escherichia*  *coli* | - | - | 10 ^4^ |
| 115 | ND | *Escherichia*  *coli* | - | - | 10 ^5^ |
| 116 | ND | *Escherichia*  *coli* | - | - | 10 ^5^ |
| 117 | ND | *Escherichia*  *coli* | - | - | 10 ^8^ |
| 118 | ND | *Escherichia*  *coli* | - | - | 10 ^7^ |
| 119 | ND | *Escherichia*  *coli* | - | - | 10 ^6^ |
| 120 | ND | *Escherichia*  *coli* | - | - | 10 ^8^ |
| NO. | Strains* | Results of VITEK 2 system | Results of PCR | Results of RealAmp | Bacterial load of sputum samples (CFU/mL ) |
| 121 | ND | *Escherichia*  *coli* | - | - | 10 ^7^ |
| 122 | ND | *Klebsiella pneumoniae* | - | - | 10 ^4^ |
| 123 | ND | *Klebsiella pneumoniae* | - | - | 10 ^8^ |
| 124 | ND | *Klebsiella pneumoniae* | - | - | 10 ^6^ |
| 125 | ND | *Klebsiella pneumoniae* | - | - | 10 ^6^ |
| 126 | ND | *Klebsiella pneumoniae* | - | - | 10 ^4^ |
| 127 | ND | *Klebsiella pneumoniae* | - | - | 10 ^7^ |
| 128 | ND | *Klebsiella pneumoniae* | - | - | 10 ^5^ |
| 129 | ND | *Klebsiella pneumoniae* | - | - | 10 ^8^ |
| 130 | ND | *Klebsiella pneumoniae* | - | - | 10 ^7^ |
| 131 | ND | *Klebsiella pneumoniae* | - | - | 10 ^6^ |
| 132 | ND | *Klebsiella pneumoniae* | - | - | 10 ^5^ |
| 133 | ND | *Klebsiella pneumoniae* | - | - | 10 ^7^ |
| 134 | ND | *Klebsiella pneumoniae* | - | - | 10 ^4^ |
| 135 | ND | *Pseudomonas maltophilia* | - | - | 10 ^5^ |
| 136 | ND | *Pseudomonas maltophilia* | - | - | 10 ^8^ |
| 137 | ND | *Pseudomonas maltophilia* | - | - | 10 ^6^ |
| 138 | ND | *Pseudomonas maltophilia* | - | - | 10 ^4^ |
| 139 | ND | *Pseudomonas maltophilia* | - | - | 10 ^8^ |
| 140 | ND | *Pseudomonas maltophilia* | - | - | 10 ^5^ |
| NO. | Strains* | Results of VITEK 2 system | Results of PCR | Results of RealAmp | Bacterial load of sputum samples (CFU/mL ) |
| 141 | ND | *Pseudomonas maltophilia* | - | - | 10 ^6^ |
| 142 | ND | *Pseudomonas maltophilia* | - | - | 10 ^7^ |
| 143 | ND | *Pseudomonas maltophilia* | - | - | 10 ^5^ |
| 144 | ND | *Pseudomonas maltophilia* | - | - | 10 ^7^ |
| 145 | *Acinetobacter baumannii* | N | - | + | ND |
| 146 | *Acinetobacter baumannii* | N | - | + | ND |
| 147 | *Acinetobacter baumannii* | N | - | + | ND |
| 148 | *Acinetobacter baumannii* | N | - | + | ND |
| 149 | *Acinetobacter baumannii* | N | - | + | ND |
| 150 | *Acinetobacter baumannii* | N | - | + | ND |
| 151 | *Acinetobacter baumannii* | N | + | + | ND |
| 152 | *Acinetobacter baumannii* | N | + | + | ND |
| 153 | *Acinetobacter baumannii* | N | + | + | ND |
| 154 | *Acinetobacter baumannii* | N | + | + | ND |
| 155 | *Acinetobacter baumannii* | N | + | + | ND |
| 156 | *Acinetobacter baumannii* | N | + | + | ND |
| 157 | *Acinetobacter baumannii* | N | + | + | ND |
| 158 | *Acinetobacter baumannii* | N | + | + | ND |
| 159 | *Acinetobacter baumannii* | N | + | + | ND |
| 160 | *Acinetobacter baumannii* | N | + | + | ND |
| NO. | Strains* | Results of VITEK 2 system | Results of PCR | Results of RealAmp | Bacterial load of sputum samples (CFU/mL ) |
| 161 | *Acinetobacter baumannii* | N | + | + | ND |
| 162 | *Acinetobacter baumannii* | N | + | + | ND |

*: The species of *Acinetobacter* determined by 16S rRNA gene sequencing.

VITEK 2 system: a fluorescence-based automated identification system.

PCR: polymerase chain reaction.

RealAmp: real-time loop-mediated isothermal amplification.

CFU/mL: colony forming units/mL.

**+**: Result of PCR or RealAmp reaction was positive.

**-**: Result of PCR or RealAmp reaction was negative.

N: No pathogenic bacteria growth.

ND: Not detected.
